# Changes in Medical Schools

**Published:** 1850-09

**Authors:** 


### CHANGES IN MEDICAL SCHOOLS.

We have still other changes to chronicle in our medical schools. Dr. Bartlett and Dr. Gross have both severed their connection with the University of Louisville and been elected to Professorships in the University of the city of New York. The former of these gentlemen accepted the chair in Louisville with the understanding that he was at liberty to give it up at any time that one should be tendered to him in an Eastern school. He had been leading, as he expressed it to a friend in this city, "a wandering and provisional sort of life for nearly ten years, and longed for a fixed *home*." Dr. Gross was induced to make the exchange by the prospect of the enlarged field for practice and literary labor afforded by the metropolis of our country. But it is not to be disguised that other considerations, growing out of the temper and policy of our municipal authorities, had their weight with both gentlemen. We need not say that their decision, especially at so late a period in the vacation, is regretted by the friends of the school, but the Board of Trustees expect in a very short time to be able to announce a satisfactory reorganization of the Faculty. Y.

